# Supplementary material for: Effects of Phycosphere Bacteria on Their Algal Host Are Host Species-Specific and Not Phylogenetically Conserved
Source: Microorganisms. 2022 Dec 25;11(1):62. doi: 10.3390/microorganisms11010062 (PMC9862884; doi:10.3390/microorganisms11010062)
Supplement: Supplementary file 1 [file microorganisms-11-00062-s001.zip › microorganisms-2120999-supplementary.pdf]

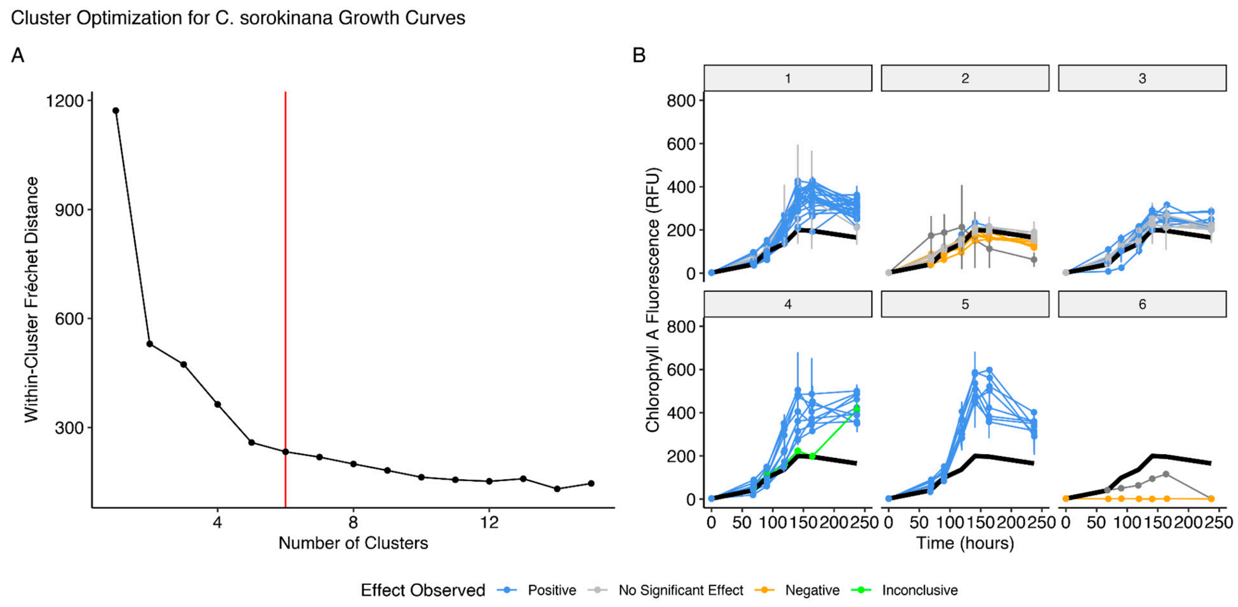

Figure S1: Clustering of isolates in preparation for cross-host algal fitness assays. (A) Within-cluster Fréchet distance for number of clusters observed. The optimal number of clusters is shown at the "elbow" of the plot in red. (B) Optimal growth curve clusters for *C. sorokiniana*. In this example, 6 types of clusters with different growth effects were detected, and a random isolate from each cluster was chosen as the representative isolate for the cross-host algal fitness assays.

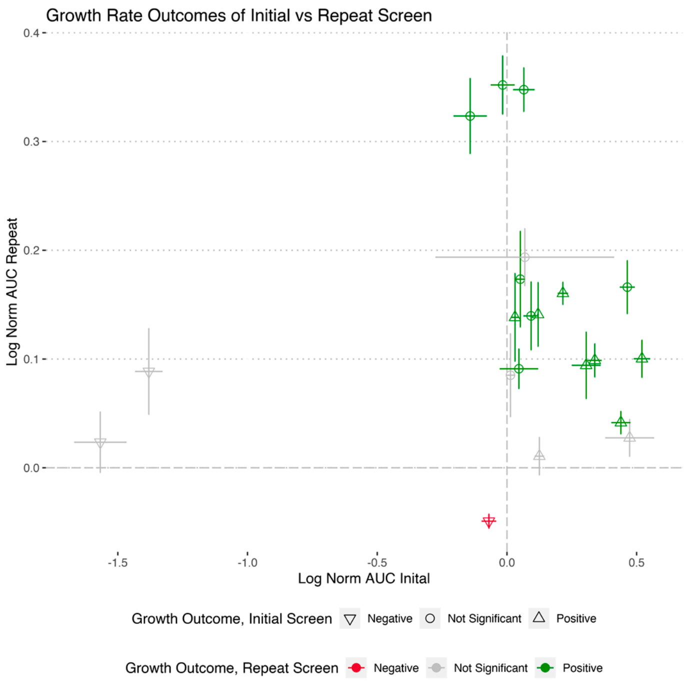

Figure S2: Replicability of host growth effects across experiments. Data is like Figure 3, except for the area under the curve data **(**AUC) comparing growth effects on the host the bacterial culture was isolated from in the initial screening experiment (horizontal axis), and the same algal-bacterial combination during the cross-host experiments (vertical axis).
